# Copper-induced activation of TRP channels promotes extracellular calcium entry, activation of CaMs and CDPKs, copper entry and membrane depolarization in *Ulva compressa*

**DOI:** 10.3389/fpls.2015.00182

**Published:** 2015-03-20

**Authors:** Melissa Gómez, Alberto González, Claudio A. Sáez, Bernardo Morales, Alejandra Moenne

**Affiliations:** ^1^Laboratory of Marine Biotechnology, Department of Biology, Faculty of Chemistry and Biology, Universidad de Santiago de ChileSantiago, Chile; ^2^Departamento de Medio Ambiente, Facultad de Ingeniería, Universidad de Playa AnchaValparaíso, Chile; ^3^Centro de Estudios Avanzados, Universidad de Playa AnchaViña del Mar, Chile

**Keywords:** ATP, copper, calcium, light, marine alga, membrane depolarization, TRP channels, *Ulva compressa*

## Abstract

In order to identify channels involved in membrane depolarization, *Ulva compressa* was incubated with agonists of TRP channels C5, A1 and V1, and the level of intracellular calcium was detected. Agonists of TRPC5, A1 and V1 induced increases in intracellular calcium at 4, 9, and 11 min of exposure, respectively, and antagonists of TRPC5, A1, and V1 corresponding to SKF-96365 (SKF), HC-030031 (HC), and capsazepin (CPZ), respectively, inhibited calcium increases indicating that functional TRPs exist *in U. compressa*. In addition, copper excess induced increases in intracellular calcium at 4, 9, and 12 min which were inhibited by SKF, HC, and CPZ, respectively, indicating that copper activate TRPC5, A1, and V1 channels. Moreover, copper-induced calcium increases were inhibited by EGTA, a non-permeable calcium chelating agent, but not by thapsigargin, an inhibitor of endoplasmic reticulum (ER) calcium ATPase, indicating that activation of TRPs leads to extracellular calcium entry. Furthermore, copper-induced calcium increases were not inhibited by W-7, an inhibitor of CaMs, and staurosporine, an inhibitor of CDPKs, indicating that extracellular calcium entry did not require activation of CaMs and CDPKs. In addition, copper induced membrane depolarization events at 4, 8, and 11 min and these events were inhibited by SKF, HC, CPZ, and bathocuproine, a specific copper chelating agent, indicating that copper entry through TRP channels leads to membrane depolarization. Moreover, membrane depolarization events were inhibited by W-7 and staurosporine, indicating that activation of CaMs and CDPKs is required to allow copper entry through TRPs. Interestingly, copper-induced calcium increases and depolarization events were light-dependent and were inhibited by DCMU, an inhibitor of photosystem II, and ATP-γ-S, a non-hydrolizable analog of ATP, suggesting that ATP derived from photosynthesis is required to activate TRPs. Thus, light-dependent copper-induced activation TRPC5, A1 and V1 promotes extracellular calcium entry leading to activation of CaMs and CDPKs which, in turn, promotes copper entry through TRP channels and membrane depolarization.

## Introduction

Transient Receptor Potential (TRP) channels are ionotropic cation channels involved in the perception of local environment, present in organisms such as humans, mammals, flies, nematodes, and yeast (Nilius and Owsianik, 2011). In mammals, TRPs are activated by stimuli such as: injury, temperature, pH, osmolarity, and pressure; by pungent compounds extracted from plants such as vanillin, capsaicin, cinnamaldehyde, allyl-isothiocyanate, camphor, menthol, cannabinoids, among others; and by inflammatory and signaling mediators such as prostaglandines, cytokines, inositol phosphates, diacylglycerol, etc. (Premkumar and Abooj, 2013). Human and mammalian TRPs are classified in 28 members belonging to six families: TRPC (Canonical), which are homologs to the first cloned TRP channel from the eye of *Drosphila melanogaster* (Montell and Rubin, 1989), include seven members that correspond to TRPC1 to TRPC7; TRPV (Vainnilloid), with six members from TRPV1 to TRPV6; TRPM (Melastatin), with eight members from TRPM1 to TRPM8; TRPML (Mucolipin), with three members from TRPML1 to TRPML3; TRPP (Polycystin), with three members from TRPP1 to TRPP3; and TRPA (Ankirin), with only one member, TRPA1 (Gees et al., 2010). The structure of TRPs is reminiscent to Voltage-Dependent Calcium Channels (VDCC), comprising six putative trans-membrane domains (S1 to S6), free intracellular N and C terminal domains, and the pore-forming loop located between S5 and S6. TRPC and TRPV family members contain domain 3 to 4 ankyrin repeats in the N terminal whereas TRPA1 contain 14 ankyrin motifs and this sequence is absent in other TRP family members (Owsianik et al., 2006). It has been shown that the ankyrin repeats allow protein-protein interactions and binding of ligands such as ATP and calmodulins (CaMs) (Gaudet, 2008).

Some TRP channels such as TRPC1/5 and TRPV5/6 are highly specific for calcium whereas others such as TRPV1 and TRPA1 are only moderately specific for calcium (Owsianik et al., 2006; Gees et al., 2010). Furthermore, the entry of extracellular calcium or other ions through TRPs leads to membrane depolarization, thus, participating in the activation of VDCC in human cells (Owsianik et al., 2006). TRPs can be modulated by intracellular signals such as binding of CaMs (Gaudet, 2008), phosphorylation by protein kinases (Yao et al., 2005), oxidation by hydrogen peroxide or nitric oxide (Takahashi et al., 2008), and by interaction with products of phospholipase C such as inositol 1, 4, 5 triphosphate (IP_3_), inositol 4, 5 biphosphate (IP_2_) and diacylglycerol (DAG) (Woo et al., 2008; Rohacs, 2013).

Human TRPs can be also activated by heavy metals as observed in TRPC5 under Pb^+2^ and Hg^+2^ (Sukumar and Beech, 2010; Xu et al., 2012), TRPA1 under Zn^+2^ (Hu et al., 2009), Cu^+2^ and Cd^+2^ (Gu and Lin, 2010), and TRPV1 under Cu^+2^, Zn^+2^, Fe^+2^ (Riera et al., 2007) and Ni^+2^ (Luebbert et al., 2010). Thus, TRPA1 and V1 are activated by Cu^+2^ and other heavy metals in human cells. In addition, it has been shown that several divalent metals cations such as Mg^+2^, Mn^+2^, Ba^+2^, Zn^+2^, Ni^+2^, Co^+2^, and Sr^+2^ can permeate different human TRP channels, including TRPA1, C5, and V1 (Bouron et al., 2014). However, until now it has not been shown that that Cu^+2^ can permeate human or animal TRP channels.

On the other hand, TRP genes have been identified in the genome of the unicellular green microalga *Chlamydomonas reinhardtii* (Wheeler and Brownlee, 2008). Recently, a functional TRP channel has been identified in *C. reinhardtii*, TRP11, which corresponds to a mechanosensitive channel located in the flagellum, involved the avoidance reaction (Fujiu et al., 2011). TRP11 is structurally related to the mammalian TRPV5 but its activity has not been observed to be inhibited by econazole. Thus, TRPs exist in microalgae and do not share some functional properties with human TRPs. It is important to mention that TRP channels are not present in plants, indicating that TRP genes might have been lost in plants during evolution. Until now, TRP channels have not been detected in marine algae.

The marine alga *Ulva compressa* (Chlorophyceae) is a cosmopolitan species tolerant to heavy metals, in particular to copper, and can be found in highly polluted coastal areas of northern Chile (Ratkevicius et al., 2003). It has been demonstrated that this alga cultivated *in vitro* with a sub-lethal concentration of copper (10 μM) showed intracellular calcium increases at 2, 3, and 12 h of copper exposure, which were due to calcium release from endoplasmic reticulum (ER) through ryanodine-, IP_3_-, and NAADP-dependent channels (González et al., 2010a,b, 2012a). In addition, copper-induced calcium increases orchestrate intracellular hydrogen peroxide and nitric oxide (NO) increases indicating there is a cross-talk among these intracellular signals (González et al., 2012a). Moreover, calcium increases induced activation of defense genes via calmodulins (CaMs) and calcium-dependent protein kinases (CDPKs) (González et al., 2012a). Furthermore, intracellular calcium increases required extracellular calcium entry through VDCC, indicating that a calcium-dependent calcium release mechanism is operating in the alga in response to copper excess (González et al., 2012b). In order to identify channels that may lead to membrane depolarization and further activation of VDCC, we analyzed the potential existence of functional TRPs in *U. compressa* that may be activated by copper leading to extracellular calcium and/or copper entry and membrane depolarization.

## Materials and methods

### Algal and seawater sampling

*U. compressa* was collected in Cachagua (32° 34′S), a non-impacted site of central Chile (Ratkevicius et al., 2003), during spring 2013 and 2014 and transported to the laboratory in sealed plastic bags in a cooler at 4°C. Algal samples were rinsed three times in sterile filtered seawater and cleaned manually. Ultrasound was applied twice for 1 min using a Branson 3200 (Danbury, CT, USA) ultrasound bath to remove epiphytic bacteria and organic debris. Seawater was obtained from the pristine site Quintay (33° 12′S) in central Chile, filtered through 0.45 and 0.2 μm pore size membrane filters and stored in darkness at 4°C.

### Treatment with agonists, antagonists, chelating agents, and copper

The agonists used in this study were purchased form Sigma (St Louis, Mi, USA): specific agonists of TRPC5 such as the lanthanides LaCl_3_ and GdCl_3_ and the signaling sphingolipid sphingosine-1-P; specific agonists of TRPA1corresponding to the flavor compounds cinnamaldehyde from cinnamon and allyl-isothiocyanate from garlic, and the heavy metal PbCl_2_; specific agonists of human TRPV1 such as the flavor compound vainillin from vanilla and the pungent compound capsaicin from Chili peppers; specific agonists of TRPM8 corresponding to the odor compound camphor from laurel tree and the flavor compound menthol from peppermint.

The antagonists of TRP channels were purchased from Sigma (St Louis, Mi, USA) and correspond to SKF-96365, a chemically synthesized inhibitor of mammalian TRPC5 (Merritt et al., 1990); HC-030031, a chemically synthesized inhibitor of mammalian TRPA1 (Eid et al., 2008), capzasepin, a synthetic analog of capsaicin that inhibits mammalian TRPV1 (Maggi et al., 1993). The fluorophor Fluo 3-AM, SBFI-AM, PBFI-AM, and Phen Green, specific for calcium, sodium, potassium, and heavy metals and copper ions, respectively, were purchased from Molecular Probes (Invitrogen, Oregon, USA); they display dissociation constants (Kd) of 350 nM, 11 mM, 10 mM, and 4 μM, respectively.

Ethyleneglycol-O, O′-bis (2-aminoethyl)-N, N, N′, N′-tetraacetic acid (EGTA), a calcium chelating agent; bathocuproine sulphonate, a specific copper chelating agent (Mohindru et al., 1983); thapsigargin, a plant sesquiterpene lactone that inhibits the endoplasmic reticulum calcium ATPase (Lytton et al., 1991); 3-(2,3-dichlorophenyl)-1,1-dimethylurea (DCMU), an inhibitor of PSII (Hsu et al., 1986); adenosine 5′-[λ-thio]triphosphate tetra-lithium salt (ATP-γ-S), a non-hydrolysable analog of ATP; N-(6-aminohexyl)-5-chloro-naphtalene sulfonamide (W-7), an inhibitor calmodulins (Hidaka et al., 1981); staurosporine, a bacterial alkaloid that binds to ATP-binding site of most mammalian protein kinases and inhibits plant calcium-dependent protein kinases (CDPKs) (Tanramluk et al., 2009) and 2-(4-carboxyphenyl)-4,4,5,5-tetramethylimidazoline-1-oxyl-3-oxide (cPTIO), a NO scavenger (Akaike et al., 1993) were also purchased from Sigma (St. Louis, Mi, USA).

For treatment with agonists, antagonists and copper, the alga was exposed to sun light for 2 h and incubated in seawater with 1 mM of agonists, 1 mM of antagonists or 250 μM copper for 15 min and the level of intracellular calcium was detected by confocal microscopy using Fluo3-AM and the level of membrane depolarization using DiOC2. It is important to point out that different concentration of agonists and copper were used to detect intracellular calcium increases due to TRPs activation (see Figure S1); the latter based in previous copper exposure (10 μM) studies where intracellular calcium increase was not detected. Inhibitors were added before copper, except in the case of 250 μM bathocuproine that was added 1 min after copper addition.

### Detection of intracellular calcium increase by confocal microscopy

Detection of calcium was performed as described by González et al. (2012b). Three lamina of *U. Compressa* were gently removed from culture media and incubated in seawater containing 20 μM Fluo-3AM (Molecular Probes, Invitrogen, Oregon, USA) during 10 min at room temperature. The laminae were washed three times in filtered seawater to remove fluorophor excess. The green fluorescence of Fluo 3 was visualized in each lamina by confocal microscopy using an Axiovert 100 confocal microscope (Carl Zeiss, Oberkochen, Germany), an emission wavelength of 488 nm produced by an argon laser and a filter of 505–530 nm. The intensity of green fluorescence and the red fluorescence of chloroplasts was quantified in each lamina using LSM510 software of the confocal microscope. The fluorescence intensity in each sample was normalized using chloroplasts autofluorescence.

### Detection of plasma membrane depolarization by confocal microscopy

Three lamina of *U. compressa* were gently removed from culture media and incubated in seawater containing 10 μM DiOC2 (Molecular Probes, Invitrogen, Oregon, USA) during 10 min at room temperature. The laminae were washed three times in filtered seawater to remove fluorophor excess. The green fluorescence of DiOC2 was visualized in each lamina by confocal microscopy using an Axiovert 100 confocal microscope (Carl Zeiss, Oberkochen, Germany), an emission wavelength of 488 nm produced by an argon laser and a filter of 505–530 nm. The intensity of green fluorescence and the red fluorescence of chloroplasts was quantified in each lamina using LSM510 software of the confocal microscope. The fluorescence intensity in each sample was normalized using chloroplasts autofluorescence.

## Results

### Functional TRPC5, A1, and V1 exist in *U. compressa*

**Figure 1:**
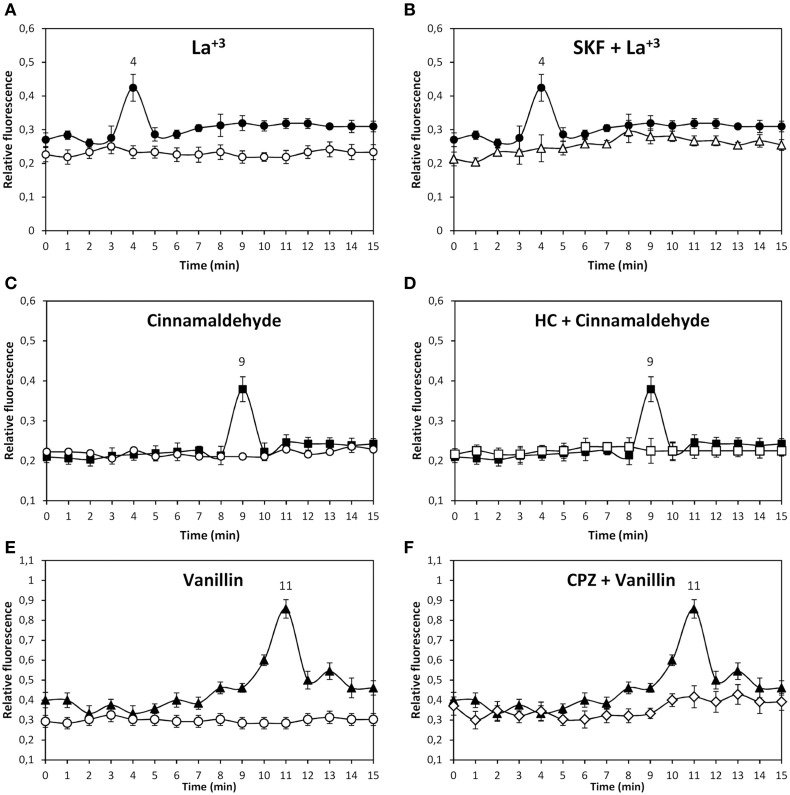
**Level of intracellular calcium in *U. compressa* cultivated in control condition (empty circles, A, C, E) or with 1 mM La^+3^ (black circles, A,B), 1 mM cinnmaladehyde (black squares C, D) and 1 mM vainillin (black triangles E, F) or with 250 μM SKF-96365 (SKF) and 1 mM La^+3^ (empty triangles, B), 250 μM HC-030031 (HC) and with 1 mM cinnamaldehyde (empty squares, D) and 250 μM capsazepin (CPZ) and 1 mM vainillin (empty diamonds, F) for 15 min**. Calcium level is expressed as the ratio of green fluorescence of Fluo 3 and red autofluorescence of chloroplasts. Symbols represent mean values of three independent experiments and ± SD.

In order to analyze the existence of functional TRP channels in *U. compressa*, the alga was loaded with the fluorophor Fluo-3AM and cultivated in control condition or with 1 m M of the specific agonists of TRPC5, La^+3^, Gd^+3^, and sphingosine-1-P, for 15 min and the increase of intracellular calcium was detected. Only La^+3^ induced an increase in intracellular calcium at 4 min of exposure (Figure 1A). In addition, the alga was incubated with 250 μM of SKF-96365 (SKF), an inhibitor of TRPC5, HC-03003 (HC), an inhibitor of TRPA1, and capzasepin (CPZ), an inhibitor of TRPV1, and with 1 mM La^+3^. The increase in intracellular calcium detected at 4 min was completely inhibited by SKF (Figure 1B) and not by HC or CPZ (data not shown). Thus, functional TRPC5 exists in the plasma membrane of *U. compressa* and its activation leads to intracellular calcium increase at 4 min of exposure.

The alga was incubated with 1 mM of the specific agonists of human TRPA1, cinnamaldehyde, allyl-isothiocyanate, and Pb^+2^, for 15 min and the increase in intracellular calcium was detected. Only cinnamaldehyde increase intracellular calcium at 9 min of exposure (Figure 1C). In addition, the alga was incubated with 250 μM of inhibitors SKF, HC and CPZ, and with 1 mM cinammaldehyde. The increase in intracellular calcium at 9 min was completely inhibited by HC (Figure 1D) and not by SKF or CPZ (data not shown). Thus, functional TRPA1 exists in the plasma membrane of *U. compressa* and its activation lead to intracellular calcium increase at 9 min of exposure.

The alga was incubated with 1 mM of the specific agonists of TRPV1 vainillin and capsaisin for 15 min and the level of intracellular calcium was detected. Vainillin induced the release of intracellular calcium at 11 min of exposure (Figure 1E) as well as capsaicin (data not shown). In addition, the alga was incubated with 250 μM of inhibitors SKF, HC and CPZ, and with 1 mM vainillin. The increase in intracellular calcium at 11 min was completely inhibited by CPZ (Figure 1F) and not by SKF or HC (data not shown). Thus, functional TRPV1 exists in the plasma membrane of *U. compressa* and its activation lead to intracellular calcium increase at 11 min of exposure.

In contrast, the specific agonists of TRPM8, menthol, and camphor, did not induce an increase in intracellular calcium at least until 30 min of agonist exposure (data not shown). Thus, TRPM8 may not be present in the plasma membrane of *U. compressa* or its activation may not lead to increase in intracellular calcium.

### Copper-induced activation of TRPs leads to extracellular calcium entry requiring ATP production but not activation of CaMs and CDPKs

**Figure 2:**
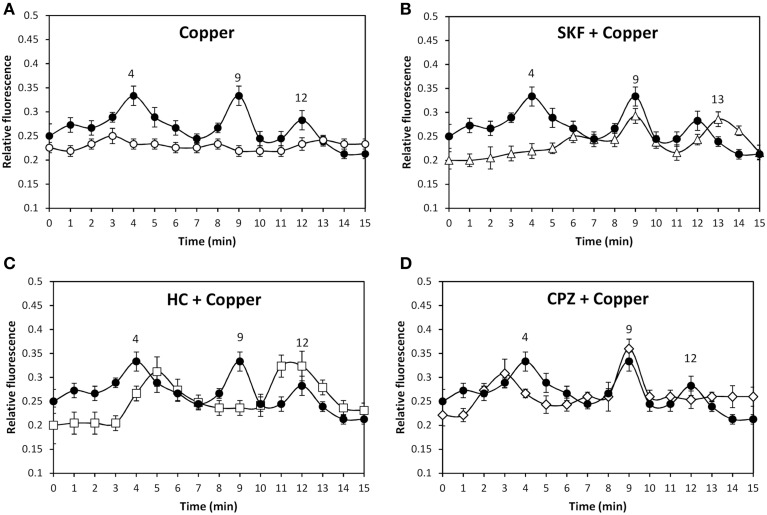
**Level of intracellular calcium in *U. compressa* cultivated in control condition (empty circles, A) and with 250 μM copper (black circles, A–D), 250 μM SKF-96365 (SKF) and 250 μM copper (empty triangles, B), 250 μM HC-03001 and 250 μM copper (empty squares, C), and 250 μM capsazepin (CPZ) and 250 μM copper (empty diamonds, D) for 15 min**. Calcium level is expressed as the ratio of green fluorescence of Fluo 3 and red autofluorescence of chloroplasts. Symbols represent mean values of three independent experiments and ± SD.

In order to analyze whether copper excess induces activation of TRP channels, the alga was loaded with the fluorophor Fluo-3AM and incubated with 250 μM copper for 15 min and intracellular calcium increase was detected. Copper induced intracellular calcium increases at 4, 9, and 12 min (Figure 2A). In addition, the alga was incubated with 250 μM SKF, HC, or CPZ and with 250 μM copper showing that the increase of intracellular calcium at 4 min was inhibited by SKF (Figure 2B), the increase at 9 min was inhibited by HC (Figure 2C) and the increase at 12 min was inhibited by CPZ (Figure 2D). Thus, copper induces activation of TRPC5, A1, and V1 channels leading to intracellular calcium increases at 4, 9, and 12 min.

**Figure 3:**
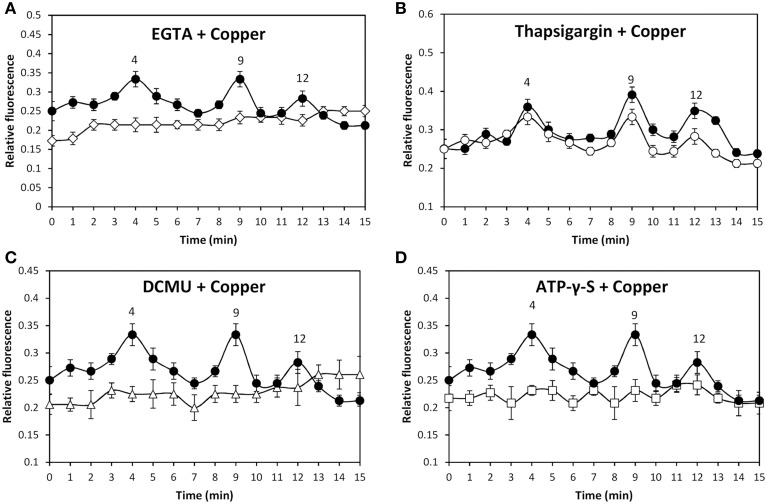
**Level of intracellular calcium in *U. compressa* cultivated with 250 μM copper (black circles, A–D), 1 mM EGTA and 250 μM copper (empty diamonds, A), 5 μM thapsigargin and 250 μM copper (empty circles, B), 100 μM DCMU and 250 μM copper (empty triangles, C), and 1 mM ATP-γ-S and 250 μM copper (empty squares, D) for 15 min**. Calcium level is expressed as the ratio of green fluorescence of Fluo 3 fluorescence and red autofluorescence of chloroplasts. Symbols represent mean values of three independent experiments and ± SD.

In order to analyze whether copper-induced activation of TRP channels leads to extracellular calcium entry and/or calcium release from internal stores, seawater was treated with 1 mM EGTA, a specific calcium chelating agent, the alga was loaded with Fluo-3AM and incubated in control and treated seawater with 250 μM copper for 15 min, and the increase in intracellular calcium was detected. EGTA completely inhibited increases of intracellular calcium at 4, 9, and 12 min (Figure 3A) indicating that intracellular calcium increases are due to extracellular calcium entry. In addition, the alga was incubated with 5 μM thapsigargin, an inhibitor of endoplasmic reticulum (ER) calcium ATPase, for 90 min (González et al., 2010b), incubated with 250 μM copper for 15 min, and the increase in intracellular calcium was detected. Thapsigargin did not inhibit copper-induced calcium increases at 4, 9, and 12 min (Figure 3B) indicating that intracellular calcium is not released from internal stores and it is exclusively due to extracellular calcium entry through TRP channels.

The alga was incubated with increasing concentrations of specific agonists of TRPC5, A1, and V1 channels or with increasing concentrations of copper in the dark showing no increases in intracellular calcium (data not shown). In contrast, exposure of the alga to the sun light for 2 h before treatment allows increases of intracellular calcium suggesting that activation of TRP channels is light-dependent. To verify the latter, the alga was exposed to sun light and incubated with 250 μM copper or with 100 μM DCMU, an inhibitor of photosystem II (PSII), and 250 μM copper for 15 min. DCMU completely inhibited the calcium increases at 4, 9, and 12 min (Figure 3C). Thus, photosynthesis is required for copper-induced activation of TRPC5, A1, and V1 in *U. compressa*. Considering that one of the main products of photosynthesis is ATP, the requirement of ATP in copper-induced activation of TRP channels was analyzed. To this end, the alga was incubated with 1 mM ATP-γ-S, a non-hydrolysable analog of ATP, and with 250 μM copper for 15 min. ATP-γ-S completely inhibited increases of intracellular calcium at 4, 9, and 12 min (Figure 3D). Thus, light-dependent ATP production is required for copper-induced activation of TRPC5, A1, and V1 in *U. compressa*.

In order to analyze whether activation of TRPC5, A1 and V1 and calcium entry are dependent on activation of phospholipase C (PLC), calmodulins (CaMs), calcium-dependent protein kinases (CDPKs) or NO production, the alga was incubated with 50 μM U73122, an inhibitor of PLC, 10 μM staurosporine, an inhibitor of plant CDPKs, 50 μM W-7, an inhibitor of CaMs and 100 μM cPTIO, a scavenger of NO and with 250 μM copper for 15 min, and intracellular calcium level was detected. The latter compounds did not inhibit intracellular calcium increases at 4, 9, and 12 min (data not shown) indicating copper-induced activation of TRP channels leading to calcium entry does not involve activation of PLC, CDPKs and CaMs or NO production.

### Copper-induced activation of TRPs leads to copper entry and membrane depolarization requiring activation of CaMs and CDPKs and ATP production

**Figure 4:**
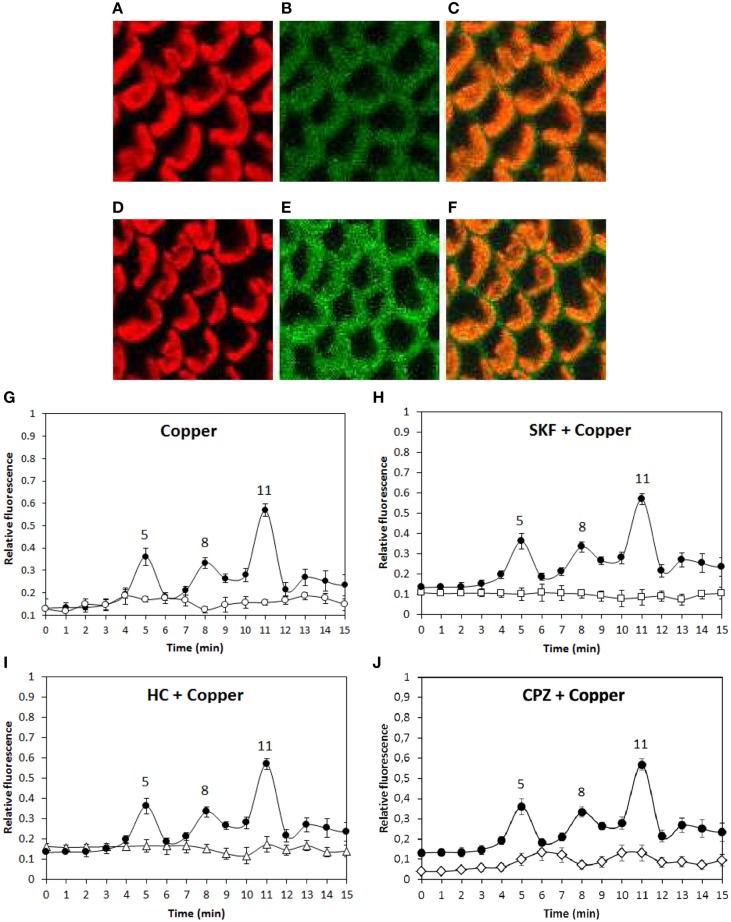
**Visualization of membrane depolarization (green fluorescence, B, E), autofluorescence of chloroplasts (red fluorescence, A, D) and merged fluorescence (C, E) in *U. compressa* exposed to 250 μM copper at 4 min of copper exposure (A–C) and at 5 min of copper exposure (D–F, first peak)**. Level of membrane depolarization in *U. compressa* cultivated in control condition (empty circles, **G**), with 250 μM copper (black circles, **G–J**), 250 μM SKF-96365 (SKF) and 250 μM copper (empty squares, **H**), 250 μM HC-030031 (HC) and 250 μM copper (empty triangles, **I**) and 250 μM capsazepin (CPZ) and 250 μM copper (empty diamonds, **J**) for 15 min. The level of membrane depolarization is expressed as the ratio of green fluorescence of DiOC2 and red autofluorescence of chloroplasts. Symbols represent mean values of three independent experiments and ± SD.

In order to analyze whether copper-induced TRPs activation leads to plasma membrane depolarization, the alga was loaded with the fluorophor DiOC2 and incubated in control condition or treated with 250 μM copper for 15 min and the increase in green fluorescence was detected (Figures 4A–F). Membrane depolarization was detected at 5, 8, and 11 min of copper exposure (Figure 4G) and these events were completely inhibited by SKF (Figure 4H), HC (Figure 4I), and CPZ (Figure 4J). Surprisingly, each inhibitor inhibited the three events of membrane depolarization which differs from results obtained for calcium entry (see above) suggesting that membrane depolarization is not due to calcium entry but instead to the entry of a different cation.

**Figure 5:**
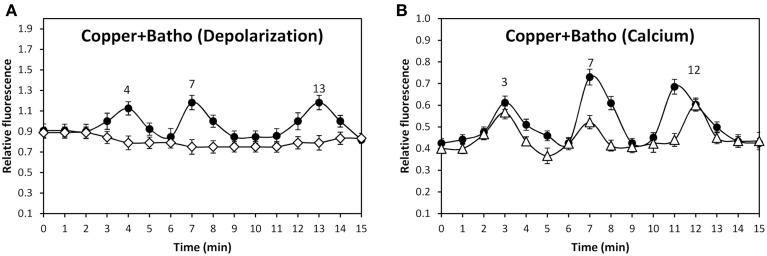
**Level of membrane depolarization (A) in *U. compressa* cultivated with 250 μM copper for 15 min (black circles) or with 250 μM copper for 1 min and then with 250 μM bathocuproine sulphonate (Batho) for 14 min (empty diamonds)**. Level of intracellular calcium **(B)** in the alga cultivated with 250 μM copper for 15 min (black circles) and with 250 μM copper for 1 min and then with 250 μM bathocuproine sulphonate for 14 min (empty diamonds). The level of membrane depolarization is expressed as the ratio of green fluorescence of DiOC2 and red autofluorescence of chloroplasts. Symbols represent mean values of three independent experiments and ± SD.

In order to identify the cation that lead to membrane depolarization, the alga was incubated with SBFI-AM, a sodium-specific fluorophor, PBFI-AM, a potassium specific fluorophor, and with Phen Green, a heavy metal-specific fluorophor, and with 250 μM copper. Surprisingly, no increases in green fluorescence were observed using the three fluorophors (data not shown). In order to detect whether copper entry may lead to membrane depolarization, the alga was incubated with 250 μM copper for 1 min and, then, 250 μM of bathocuproine sulphonate, specific copper-chelating agent, were added and membrane depolarization was detected. Membrane depolarizations events detected at 4, 7, and 13 min were completely inhibited by bathocuproine sulphonate (Figure 5A). In addition, the same experiment was performed using Fluo 3-AM to detected calcium increases. In this case, bathocuproine did not inhibit copper-induced calcium increases observed at 3, 7, and 12 min (Figure 5B). Thus, copper-induced membrane depolarization events are mainly due to copper ions entry through TRPC5, A1, and V1 channels.

**Figure 6:**
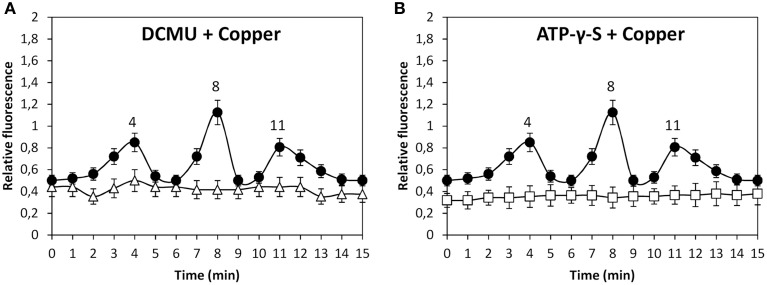
**Level of membrane depolarization in *U. compressa* cultivated with 250 μM copper (black circles, A,B), 100 μM DCMU and 250 μM copper (empty triangles, A) and with 1 mM ATP-γ-S and 250 μM copper (empty squares, B) for 15 min**. The level of membrane depolarization is expressed as the ratio of green fluorescence of Fluo 3 and red autofluorescence of chloroplasts. Symbols represent mean values of three independent experiments and ± SD.

**Figure 7:**
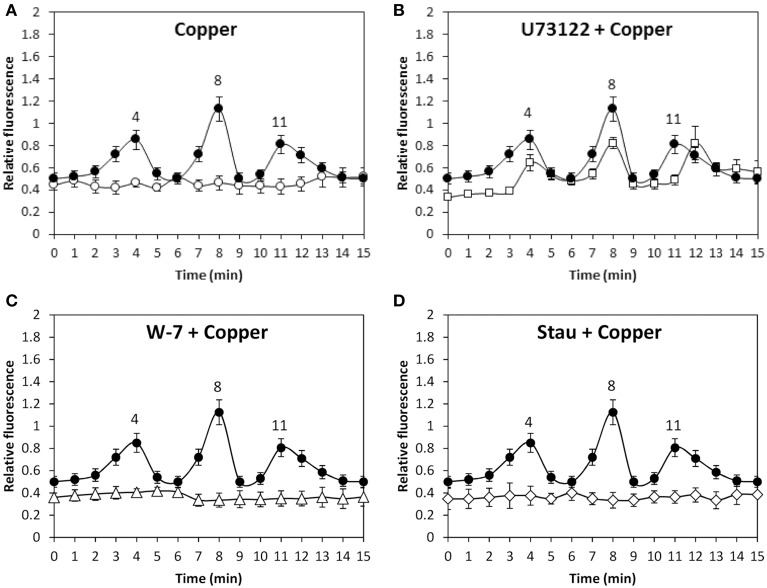
**Level of membrane depolarization in *U. compressa* cultivated in control condition (empty circle, A), with 250 μM copper (black circles, A–D), with 50 μM U73122 and 250 μM copper (empty, squares, B), with 50 μM W-7 and 250 μM copper (empty triangles, C) and 5 μM staurosporine (Stau) and 250 μM copper (empty diamonds, D) for 15 min**. The level of membrane depolarization is expressed as the ratio of green fluorescence of DiOC2 and red autofluorescence of chloroplasts. Symbols represent mean values of three independent experiments and ± SD.

In addition, copper-induced TRP-dependent depolarizations at 4, 8, and 11 min were inhibited by DCMU (Figure 6A) and ATP-γ-S (Figure 6B). Thus, copper ions entry leading to membrane depolarization requires light-dependent ATP production. Moreover, the alga was cultivated with 50 μM U73122, 50 μM W-7, 10 μM staurosporine or with 250 μM copper for 15 min, and membrane depolarization was detected. Membrane depolarization events at 5, 8, and 11 min (Figure 7A) were not inhibited by U73112 (Figure 7B) but they were completely inhibited by W-7 (Figure 7C) and staurosporine (Figure 7D). Thus, copper entry leading to membrane depolarization requires activation of CaMs and CDPKs, but not PLC activation.

## Discussion

### Functional TRPC5, A1, and V1 exist in the marine alga *U. compressa*

In this work, we showed that functional TRPC5, A1, and V1 exist in the plasma membrane of marine alga *U. compressa*. In particular, specific agonists of human TRPs activate *Ulva* TRPs as in the case of La^+3^ and TRPC5, cinnamaldehyde and TRPA1, vainillin and capsaicin and TRPV1. However, some agonists of human TRPs did not activate *Ulva* TRPs as in the case of Pb^+2^, sphingosine-1-P and TRPC5, and allyl-isothiocyanate and TRPA1, which indicates that algal TRPs are not functionally identical to human TRPs. These findings are in accord with the fact that TRP11 of *C. reinhardtii*, a unicellular green microalga, which share structural homology with TRPV5, was not inhibited by econazole, an inhibitor of human TRPV5 (Fujiu et al., 2011). In addition, activation of algal TRPC5, A1 and V1 lead to intracellular calcium increases which is in accord with the fact that human TRPC5, A1, and V1 allow calcium entry (Owsianik et al., 2006). In contrast, agonists of TRPM8 did not induce intracellular calcium increase suggesting that this TRP channel may not be present in the plasma membrane of *U. compressa* or that its activation did not lead to calcium entry. Thus, functional TRPC5, A1, and V1 exist in a green marine alga leading to intracellular calcium increases and algal TRPs share some functional properties with human TRPs. It is important to mention that calcium entry through TRP channels triggered by specific agonists did not induce membrane depolarization (data not shown), which is in accord with our observations showing that membrane depolarization occurs mainly due to copper ions entry (see below).

In addition, it is important to mention that marine algae arose on earth around 1000 million of years ago and terrestrial plants around 480 million years (Yoon et al., 2004). Considering that *U. compressa* contain functional TRPs, it is possible to conclude that TRPs were already present in the ancestors of the green linage and that they were lost in terrestrial plants. In addition, TRP genes are encoded in the genome of the green microalga *C. reinhardtii* and at least one of them, TRP11, is functional (Wheeler and Brownlee, 2008; Fujiu et al., 2011). On the other hand, it has been shown that genomes of choanoflagelates, which are unicellular ancestors of the animal lineage, also contain genes encoding seven families of TRPs (Cai, 2008). Therefore, functional TRP channels exist in cells of the green and non-green lineage before multicellular organisms arose on earth.

### Copper-induced activation of TRPs allow extracellular calcium entry and requires ATP production but not activation of CaMs and CDPKs

**Figure 8:**
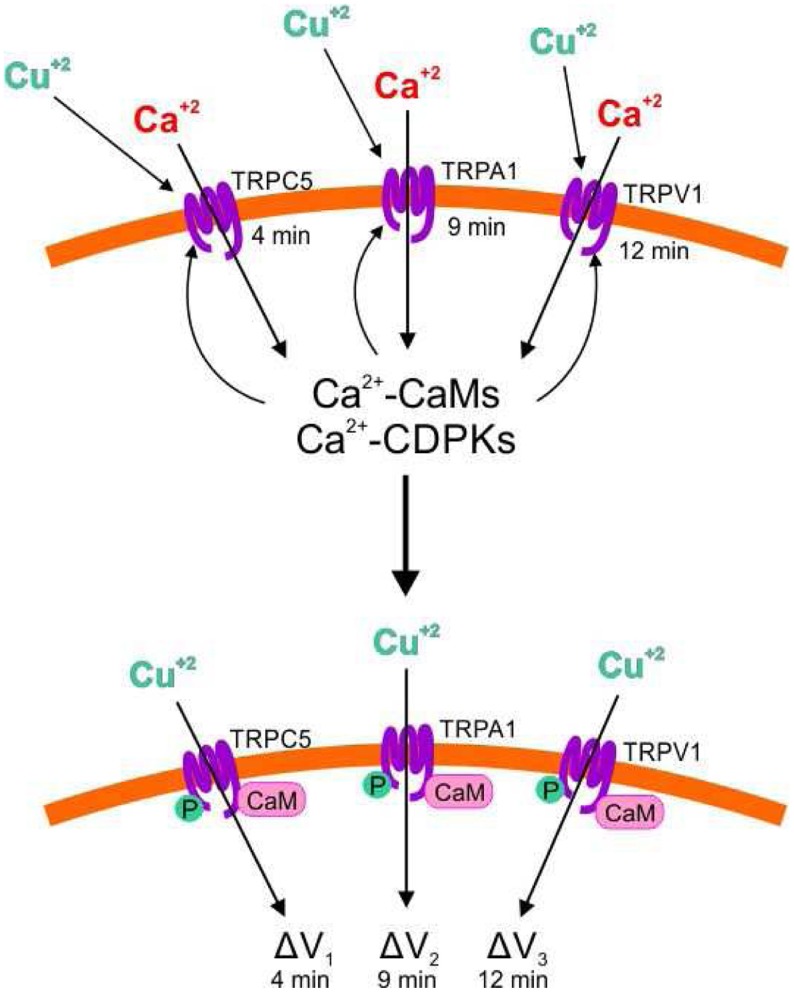
**Model of copper-induced activation of TRPC5, A1 and V1 channels leading to extracellular calcium entry, activation of CaMs and CDPKs by calcium, activation of TRPC4, A1 and V1 by CaMs and CDPKs, and extracellular copper entry leading to membrane depolarization**. Copper ions (Cu^+2^); calcium ions (Ca^+2^); Transient Receptor Potentials (TRPs); calmodulins (CaMs), calcium-dependent protein kinases (CDPKs); phosphorylation (P); membrane depolarization (ΔV).

Our results showed that copper excess induced the activation of TRPC5, A1, and V1 channels triggering extracellular calcium entry. In this sense, human TRPA1 and V1 are also activated by copper ions (Riera et al., 2007; Gu and Lin, 2010) indicating that human and *Ulva* TRPA1 and V1 share some regulatory properties. In addition, copper induced extracellular calcium entry due to activation of activation of TRPs, not involving activation of PLC, CaMs, and CDPKs or increase in NO, which suggests that TRP channels are directly activated by copper ions (see model in Figure 8), however this assumption remain to be experimentally determined. Interestingly, addition of the copper-chelating agent bathocuproine sulphonate after 1 min of copper exposure did not inhibit calcium increases, which suggest that 1 min is sufficient to ensure copper binding to TRPs leading to their activation and further calcium entry.

Moreover, we showed that photosynthesis and one of its main products, ATP, are required for the copper-induced activation of TRPC5, A1, and V1 in *U. compressa*. This finding is quite unique since mammalians, invertebrates and yeast do not undergo photosynthesis and TRPs are not present in terrestrial plants. TRP genes have been detected in the genome of the green microalga *C. reinhardtii* (Wheeler and Brownlee, 2008) and at least one of them is functional (Fujiu et al., 2011); however, until now it has not been shown that *C. reinhardtii* TRPs requires photosynthesis or ATP. In spite of the latter, it has been shown that human TRP bind CaMs and ATP but they are also subjected to phosphorylation by CDPKs (Yao et al., 2005; Gaudet, 2008). It is important to mention that copper-induced depolarization events as well as calcium increases varies in timing in different experiments which may be due to the fact that *U. compressa* collected in the field is not clonal and, thus, not physiologically identical.

### Copper-induced activation of TRPs allows copper ions entry and membrane depolarization requiring ATP production and activation of CaMs and CDPKs

Copper induced membrane depolarization occurred at 5, 8, and 11 min and they were inhibited by SKF, HC, and CPZ, indicating that these depolarizations involve activation of TRPC5, A1, and V1. Surprisingly, depolarizations at 5, 8, and 11 min were completely inhibited by W-7 and staurosporine, indicating that these depolarizations require the activation of CaMs and CDPKs by calcium that enters through TRP channels. In addition, it was shown that bathocuproine completely inhibited membrane depolarizations indicating that copper ions permeate TRPs inducing membrane depolarization (see model in Figure 8). Until now, there is no evidence that copper could permeate human or animal TRPs (Bouron et al., 2014), but this could be found in the future for human TRPs. It is important to mention that copper ions entry was not detected by the heavy metal-sensing fluorophor Phen Green (data not shown), which could be due to the lower affinity of Phen Green for copper (Kd 4 μM, see Materials and Methods) compared with that of Fluo 3-AM for calcium (Kd 350 nM). Another possibility is that copper ions are rapidly chelated inside the cells probably by phytochelatins, which have been observed to be constitutively synthesized in *U. compressa* (Mellado et al., 2012). In addition, it is not ruled out that sodium or potassium ions may also enter through TRP channels and that they were not detected by fluorophors SBFI-AM and PBFI-AM, respectively, since the latter have a lower affinity for their specific ions (Kd around 10 mM, see Materials and Methods) than Fluo 3-AM. In addition, copper entry through TRP channels required light-dependent ATP production, probably because ATP is required for phosphorylation of TRPs by CDPKs (see model in Figure 8), but this assumption remain to be determined. Finally, it is possible that copper-induced membrane depolarization of TRPs may participate in a direct or indirect manner in further activation of VDCC, but this assumption remain also to be further analyzed.

## Conclusions

In conclusion, copper activate TRPC5, A1, and V1 channels promoting extracellular calcium entry which activates CaMs and CDPKs which, in turn, regulate TRPs leading to copper ions entry and membrane depolarization which may directly or indirectly contribute to further activation of VDCC. In addition, copper-induced activation of TRPs requires light-dependent ATP production probably for TRPs phosphorylation by CDPKs.

### Conflict of interest statement

The authors declare that the research was conducted in the absence of any commercial or financial relationships that could be construed as a potential conflict of interest.
