# Supplementary figures and images for: Copper-induced activation of TRP channels promotes extracellular calcium entry, activation of CaMs and CDPKs, copper entry and membrane depolarization in Ulva compressa
Source: Front Plant Sci. 2015 Mar 20;6:182. doi: 10.3389/fpls.2015.00182 (PMC4367172; doi:10.3389/fpls.2015.00182)

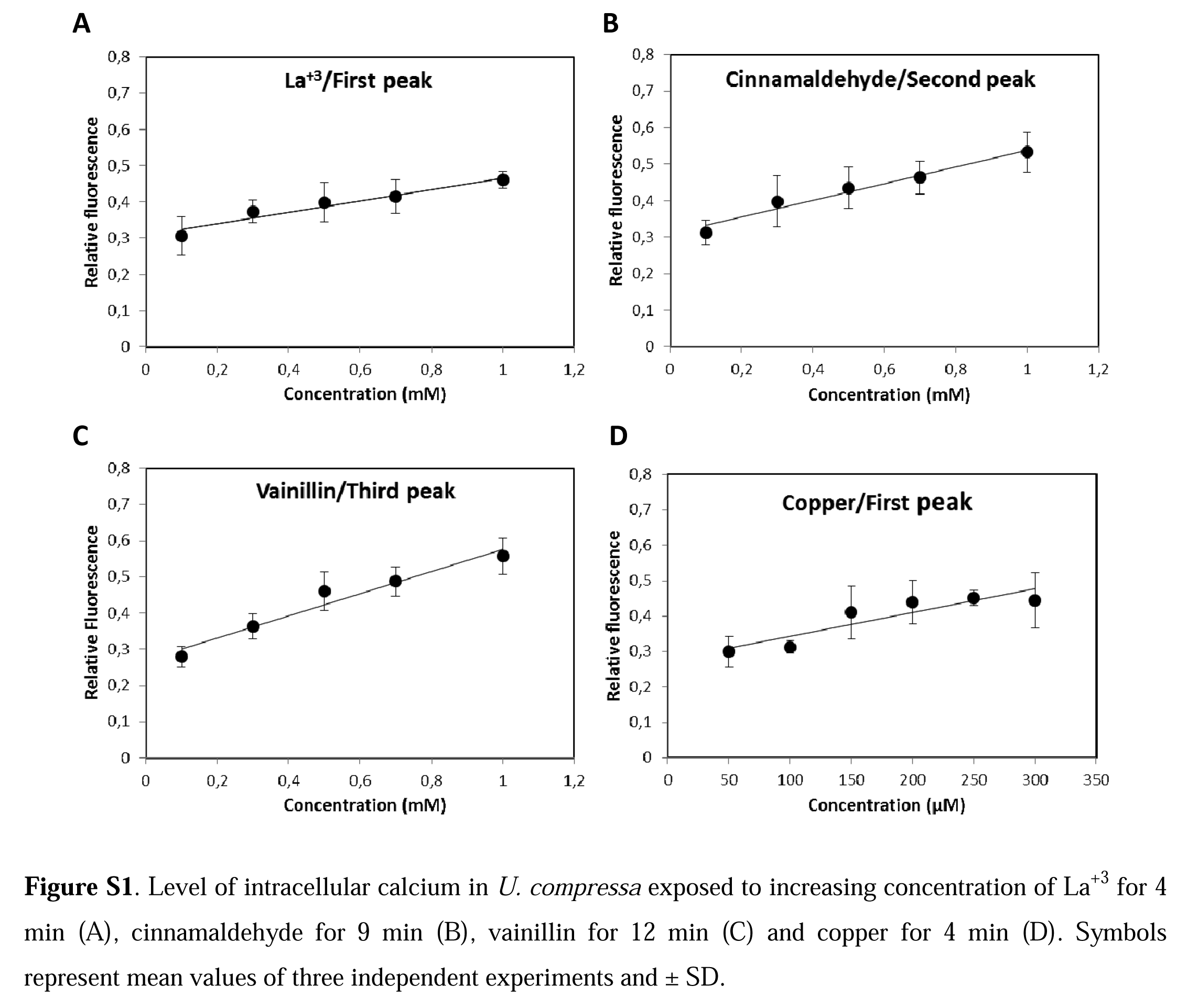

Supplement: Supplementary file 1 [file Image1.TIF]
